# Antenatal care in Nepal: a qualitative study into missed opportunities in the first trimester

**DOI:** 10.1016/j.xagr.2022.100127

**Published:** 2022-11-04

**Authors:** Felicity Greenfield, Mary Lynch, Nashna Maharjan, Miriam Toolan, Katie Barnard, Tina Lavender, Michael Larkin, Nisha Rai, Meena Thapa, Deborah M. Caldwell, Christy Burden, Dharma S. Manandhar, Abi Merriel

**Affiliations:** 1 Bristol Medical School, University of Bristol, Bristol, United Kingdom (Ms Greenfield); 2 Academic Women's Health Unit, Bristol Medical School, University of Bristol, Bristol, United Kingdom (Ms Lynch and Barnard, Drs Toolan, Burden and Merriel); 3 North Bristol NHS Trust, Bristol, United Kingdom (Ms Lynch, Barnard); 4 Mother and Infant Research Activities, Kathmandu, Nepal (Ms Maharjan, Dr Manandhar); 5 National Institute for Health and Care Research Bristol Biomedical Research Centre, Bristol, United Kingdom (Drs Burden, Toolan); 6 Liverpool School of Tropical Medicine, Liverpool, United Kingdom (Dr Lavender); 7 Department of Psychology, Aston University, Birmingham, United Kingdom (Dr Larkin); 8 Hetauda Hospital, Hetauda, Nepal (Dr Rai); 9 Kathmandu Medical College, Kathmandu, Nepal (Dr Thapa); 10 Population Health Sciences, Bristol Medical School, University of Bristol, Bristol, United Kingdom (Dr Caldwell); 11 Centre for Women's Health Research, Department of Women's and Children's Health, Institute of Life Course and Medical Sciences, Faculty of Health and Life Sciences, University of Liverpool, Liverpool Women's Hospital, Liverpool, United Kingdom (Dr Merriel)

**Keywords:** focus groups, folic acid, low-income countries, pregnancy care, South Asia, ultrasound scanning

## Abstract

**BACKGROUND:**

Use of timely antenatal care has been identified as key to facilitating healthy pregnancies worldwide. Although considerable investment has been made to enhance maternal health services in Nepal, approximately one-third of women do not attend antenatal care until after the first trimester (late). These women miss out on the benefits of screening and interventions that are most effective in the first trimester.

**OBJECTIVE:**

This study aimed to identify the missed opportunities of women who do not attend antenatal care in the first trimester, and to explore some of the factors underlying late attendance and consider potential solutions for minimizing these missed opportunities in the future.

**STUDY DESIGN:**

This study was conducted in 3 hospitals in Nepal. Focus groups (n=18) with a total of 48 postnatal women and 49 staff members, and 10 individual interviews with stakeholders were conducted. Purposive sampling facilitated the obtainment of a full range of maternity experiences, staff categories, and stakeholder positions. Data were qualitative and analyzed using a thematic approach.

**RESULTS:**

Limited awareness among women of the importance of early antenatal care was reported as a key factor behind attendance only after the first trimester. The family and community were described as significant influencers in women's decision-making regarding the timing of antenatal care. The benefits of early ultrasound scanning and effective supplementation in pregnancy were the major missed opportunities. Increasing awareness, reducing cost, and enhancing interprofessional collaboration were suggested as potential methods for improving timely initiation of antenatal care.

**CONCLUSION:**

Limited awareness continues to drive late attendance to antenatal care after the first trimester. Investment in services in the first trimester and community health education campaigns are needed to improve this issue and enhance maternal and neonatal outcomes.

**AJOG Global Reports at a Glance**

**Why was this study conducted?**

At least one-third of women in Nepal do not attend antenatal care in the first trimester. We aimed to explore the reasons for this, understand the consequent missed opportunities, and identify solutions for improving attendance.

**Key findings**

Nepali women are missing out on the benefits of early ultrasound scanning and effective supplementation in pregnancy. They and their communities have limited awareness of the importance of antenatal care in the first trimester.

**What does this add to what is known?**

Timely initiation of antenatal care could be improved using community interventions to target limited awareness, utilizing the influence of the family and the community. Addressing limited awareness, reducing cost, and enhancing interprofessional collaboration could minimize missed opportunities and improve pregnancy outcomes.

## Introduction

Antenatal care (ANC)—the routine care of pregnant women between conception and the onset of labor—has made a significant contribution to the improvement of maternal and newborn health outcomes in Nepal. According to the 2016 Nepal Demographic and Health Survey (NDHS),^1^ the maternal mortality rate has fallen from 539 maternal deaths per 100,000 live births to 239 maternal deaths per 100,000 live births between 1996 and 2016. Similarly, the neonatal mortality rate reduced during this period from 50 deaths per 1000 live births in 1996 to 21 deaths per 1000 live births in 2016.^1^

ANC enables the screening and management of pregnancy-related complications, facilitates health education, and increases births in health facilities.^2^^,^^3^ It plays a pivotal role in the World Health Organization's (WHO) ambition for a world where “every pregnant woman and newborn receives quality care throughout the pregnancy, childbirth and postnatal period.”^4^

The quality of ANC is determined by 3 key elements: the timing of initiation of care, the number of visits, and the inclusion of the recommended components of care.^5^ The WHO increased the number of contacts in its recommended ANC model from 4 to a minimum of 8 in 2016, and this new model was recently adopted in Nepal, after the time of this study.^6^ In this model, the first contact should be in the first 12 weeks of pregnancy. However, only two-thirds of pregnant women attended ANC in the first trimester according to the 2016 NDHS.^1^ Therefore, approximately one-third of women miss out on the benefits and opportunities that ANC in the first trimester affords. These include several screening programs (eg, ultrasound scanning) and intervention programs (eg, dietary supplements) that are most effective when performed early.^7^ Findings from a recent service evaluation by our group^8^ differed from those of the recent NDHS.^1^ We found that only 22% of women received ANC in the first trimester. This study, set in the same sites as the service evaluation, aimed to identify the missed opportunities faced by women who attend ANC only after the first trimester. It aimed to explore the factors behind this late attendance and consider potential solutions for minimizing these missed opportunities in the future.

## Methods

### Setting

The study was based at 3 hospitals in Nepal. These were chosen purposively to represent the different parts of the health system and achieve a broad understanding of currently available ANC. These included a tertiary referral hospital and a private secondary care teaching hospital in Kathmandu, and a district secondary care hospital in Makwanpur, with approximately 19,000, 3600 and 2500 annual births, respectively.

### Participants

Sampling for focus groups and interviews was done purposively to achieve a wide range of perspectives. This included a range of maternity experiences (first-time mothers and women in subsequent pregnancies, births with and without complications), staff categories (doctors of different seniority, nurses, midwives, nontrained nursing staff, and community health volunteers), and stakeholder positions (from governmental and nongovernmental organizations such as the Department of Health Services and the Nepal Society of Obstetricians and Gynaecologists). This range of perspectives was essential for considering potential solutions for minimizing missed opportunities caused by not attending ANC in the first trimester. Speaking to women with diverse experiences was important because maternity services need to effectively deliver care to women across the spectrum of parity and risk. All cadres of healthcare staff and stakeholders provide important perspectives to improving care. However, not all staff groups were available on the days when the research team visited the sites; for example, in the secondary hospital, no doctors were available for interview (Table 1). To remedy this, we ensured that we interviewed a stakeholder from the secondary hospital to obtain their perspective.

**Table 1:** Staff focus groups

Characteristics	Private hospital	Secondary hospital	Referralhospital	Overall
	n=11	n=17	n=21	n=49
Age (mean, y)	29.0	34.9	37.5	33.8
Years of professional work (mean)	5.0	12.0	14.7	10.6
Doctors	11	0	11	22
Nurses	0	5	5	10
Auxiliary nurse midwives	0	7	5	12
Female community health volunteers	0	5	0	5

The number of focus groups was determined pragmatically to ensure representation across the sites, patients, and staff groups. Three focus groups were conducted for postnatal women at each site, with up to 5 participants in each; 22% of the postnatal women surveyed at our study sites had attended ANC in their first trimester.^8^ We considered that having a mixture of women who attended and who did not attend ANC in the first trimester would assist in addressing our research question by highlighting factors behind the timing of first ANC attendance.

Staff and women were recruited in person by 2 experienced qualitative research assistants with master's degrees in sociology who verbally explained the study to them. Women were recruited from the postnatal ward or at their postnatal review. They were informed that the study would explore their perceptions regarding the ANC they received in their latest pregnancy. Staff groups were formed according to their different roles to encourage participation, and 3 focus groups of staff were held at each site. Ten stakeholders were recruited via telephone, and then one-on-one in-person interviews were conducted by the same 2 research assistants. Participants were informed about the research details in the morning, and the focus groups were conducted in the evening. Written consent was obtained via fingerprint or signature. No one approached by the research team refused to participate or dropped out.

### Research team

The study was conducted in Nepal by a research nurse and doctor (N.M., D.S.M.), both with extensive experience in conducting maternal health research in Nepal. The focus group discussions were moderated by the same 2 research assistants who recruited participants. In the United Kingdom, the study was led by an obstetrician (A.M.) with experience in qualitative research in low- and middle-income countries, and supported by a wider research team including midwives, psychologists, and doctors.

### Data collection

Data collection occurred between February and April of 2019 either simultaneously or over a short period in all sites, rather than by sample groups feeding into each other. The field researchers conducted mock interviews and focus group discussions before this period. The women's focus groups, conducted in postnatal hospital wards, began with a short demographic survey of each woman, followed by approximately 60 minutes of discussion. They were moderated by 2 members of the research team, one leading the questioning and the other taking field notes. This method was repeated in hospital staff rooms for staff focus groups. A Topic Guide (Supplemental File 1) was used to prompt discussion. Semistructured interviews with stakeholders in their offices were carried out by one researcher (N.M.) with a specific, altered topic guide. Other nonparticipants, except for the mothers’ infants, were not present during discussions. All participants were asked questions about ANC generally in Nepal and their ideas on how it could be improved. The interviews were transcribed verbatim, anonymized, and translated into English.

### Data analysis

Initial thematic analysis^9^ was carried out by the core research team, who were a multidisciplinary group of a nurse, a midwife, and 2 doctors (N.M., M.T., M.L., A.M.). A further in-depth analysis of this specific area was then carried out by this team alongside a medical student (F.G.). The steps that were involved in the analysis process are described in Table 2.

**Table 2:** Steps in data analysis

Braun and Clarke's 6 steps of thematic analysis	How these were applied in this study
1. Familiarization	The interviews were transcribed verbatim, anonymized, and translated into English by the MIRA research team in Nepal. The core research team read the transcripts and noted initial ideas, including the idea of missed opportunities in the first trimester.
2. Generating initial codes	Systematic coding was carried out across the whole data set by the core research team. Then, it was coded using the predetermined lens of missed opportunities in the first trimester by F.G. and M.L. separately. Each transcript was coded by at least 2 people independently.
3. Searching for themes	F.G. and M.L. separately collated codes into potential themes. This was done manually. F.G. initially sorted codes into the missed opportunities identified, the reasons for these, and ideas for improvement (Supplemental File 3). Around these 3 core categories, the diagram includes relevant codes that had been mentioned multiple times in the data.
4. Reviewing themes	Themes were discussed by the core research team and F.G., producing a basic thematic map.
5. Defining and naming themes	Themes were refined by the same team and theme names were generated using key quotes from each theme (Figure).
6. Producing the report	Representative quotes were selected for the Results section. Additional illustrative quotes were selected (Table 5). These were related to the literature on delayed attendance to ANC and facilitated the discussion and conclusion.

*ANC*, antenatal care; *MIRA*, Mother and Infant Research Activities.

## Results

Overall, 49 staff members and 48 women took part in 18 focus groups (Tables 1 and 3), and 10 stakeholders Table 4 were individually interviewed, ranging from district- to national-level involvement. The mean age of the postnatal women across the 3 hospitals was 25.9 years (Table 3). This younger age is reflective of Nepal's general median age at first birth of 20.4 years (according to the 2016 NDHS),^1^ with our study including a mixture of primiparous and multiparous women.

**Table 3:** Women's focus groups

Characteristics	Private hospital	Secondary hospital	Referral hospital	Overall
	n=14	n=19	n=15	n=48
Age (mean, y)	26.8	25.0	25.8	25.9
Standard deviation of age	3.2	4.0	4.9	4.1
% (n) primiparas	50 (7)	47 (9)	60 (9)	52 (25)
% (n) multiparas	50 (7)	53 (10)	40 (6)	48 (23)

**Figure 1:**
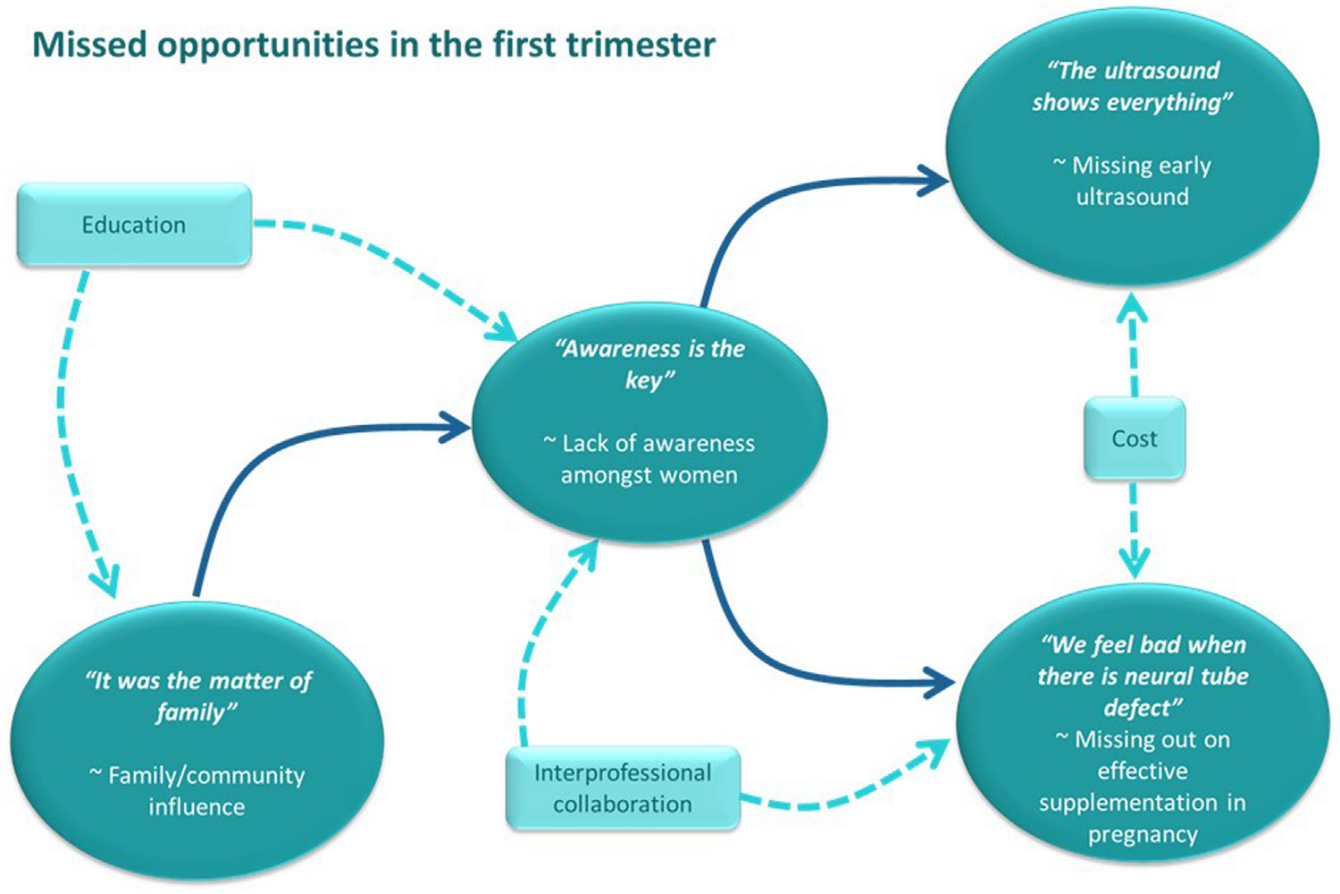
Thematic map Thematic map showing the connections between the 4 themes (*oval shapes*) and some potential interventions (*blue boxes*) identified from discussions.

We refined the data into four key themes, developed from the initial thematic map (supplementary file 3). These were encapsulated best by the words of participants: “Awareness is the key”; “It was the matter of family”; “The ultrasound shows everything”; and “We feel bad when there is neural tube defect.” These themes are presented in Figure 1 with illustrative quotes in Table 5 and are discussed below.

Greenfield. Missed opportunities from late antenatal care in Nepal. Am J Obstet Gynecol Glob Rep 2022.

When considering the themes, it was apparent that they were all interlinked, and limited awareness was the central mechanism for these interactions.

### “Awareness is key”

“…They have this idea that they should visit only after they are 3 months pregnant.” (doctor in referral hospital – P2, FGD2). Consistent feedback from staff and stakeholders described a limited awareness among women of the importance of ANC in the first trimester. This was reinforced by women's focus groups that discussed limited knowledge as a barrier to attendance: “…the main reason for this is lack of education… If women had known that they should go for the check-up, then they would have done it.” (woman in secondary hospital – P3, FGD 3).

Staff explained a common opinion that there was no need to visit health facilities in the first trimester—a misconception that was reinforced by “everybody around the pregnant woman” (doctor in referral hospital – P3, FGD2).

Some women described not attending ANC in the early weeks because they were not aware that they were pregnant, whereas others were aware but were unable to attend because of other responsibilities.

“…How could I know that there was a baby? After that I came here, and when I consulted with the doctor I was told that there is a baby of 3 months... That is why there was delay in seeking care.” (woman in private hospital – P2, FGD1).

“Although I had a plan to go to Kathmandu (for ANC early in pregnancy), I could not go because of housework…” (woman in referral hospital – P4, FGD2).

Poor understanding of the free services available and perceived costs also prevented ANC uptake in the first trimester.

“…had they (pregnant women) known that everything is for free since the very beginning, more people would undoubtedly visit us…” (doctor in referral hospital – P4, FGD2).

Staff noted recent improvements in awareness among women, resulting in earlier visits to hospitals. However, they also highlighted the need for further improvement, and specifically for challenging the misconception that ANC should be initiated after 16 weeks.

“We advise patients to visit as soon as you know you are pregnant in the first trimester, so that we can provide folic acid. But most patients visit only at 16 weeks. Even women from educated, urban areas visit at 16 weeks. When asked why, they said that this advice was provided by the media and that this is the government's policy…” (Stakeholder 3).

Improving awareness was identified as key in improving uptake of ANC in the first trimester and minimizing missed opportunities.

### “It was a matter of family”

During the doctors’ and postnatal women's focus groups, many participants suggested that pregnant women's family, friends, and community were key to influencing their decision on when to initiate ANC. All the women in FGD1 in the referral hospital said they took the advice of their husband on when to attend ANC. They also described their mothers or mothers-in-law advising that ANC was not necessary in early pregnancy, justifying it by the fact that they had not had any ANC in their pregnancies.

“Because mothers know everything about the pregnancy, what are the symptoms, when to worry, when not to and what should I eat and what not to. I was suggested by my mother for five month and after that I was suggested by the doctor.” (Woman in referral hospital – P1, FGD1).

Limited awareness within the community was reported, solidifying the misconception that ANC should only be initiated after the first trimester. Intervention by involving the community in education programs was suggested to boost early initiation of ANC.

“…they say that their near ones only came when they were 3 months pregnant... Women, instead of deciding themselves and using logic, follow others’ advice. And the ones who are giving advice are giving wrong advice. This is the reason why the society as a whole needs to be made aware.” (doctor in referral hospital – P4, FGD2).

### “the ultrasound shows everything”

Staff in all 3 hospitals emphasized the missed opportunity of the benefits of early ultrasound when women initiate ANC after the first trimester. Many women failed to receive ANC in the first trimester and ultrasound scans within 24 weeks (Table 6).

“There are also some cases in which after ultrasound, it is found that the baby does not have a head. If at least these visits are made, that very problem can be avoided.” (doctor in referral hospital – P6, FGD2).

Limited awareness of the importance of first-trimester ultrasound scanning among pregnant women was described as a key factor behind delays in scanning. However, poor availability of ultrasound and an inadequate capacity in hospitals to manage the high numbers of patients were also mentioned as barriers by staff, stakeholders, and women.

“The women only come after the completion of 4 months. And there is a delay in providing the right services to them. The ultrasound is provided only after 2 to 3 months.” (female community health volunteer in secondary hospital – P2, FGD1).

“The services provided for ultrasound aren't good. We have to wait for the time.” (woman in private hospital – P3, FDG 2).

The importance of ultrasound scanning to know the condition of their infant was frequently mentioned in the focus groups of postnatal women. This supported staff and stakeholder opinions that ultrasound services attracted women to ANC and that making them free could encourage women to use this service.

“The ultrasound shows everything. It shows about the development of the baby, growth of the baby, weight…” (woman in secondary hospital – P5, FGD1).

“…Women would be more likely to go for a visit if they knew that they could have ultrasound and blood tests as well…” (Stakeholder 1).

### “We feel bad when there is neural tube defect”

Both women and staff reported that effective supplementation is rarely achieved. They described the provision of a combined tablet of iron and folic acid from the second trimester of pregnancy. The timing of folic acid supplementation is crucial, ideally beginning preconceptually and continuing until 12 weeks of gestation. This assists in forming the neural tube during early development and prevents major birth defects such as spina bifida.^10^ Doctors and stakeholders reported concerns about this missed opportunity of benefits of effective folic acid supplementation and the rarity of preconceptual care. Neural tube disorders were described in discussions to be “increasing” and “very high.”

“We have to provide folic acid supplementation... within 13 weeks. And this is not provided to the patients. We feel bad when there is a neural tube defect for which we don't have medicines.” (doctor in private hospital – P2, FGD2).

Staff and stakeholders noted cost as a barrier to uptake, similar to ultrasound scanning. Limited awareness was also key because women were unaware of the importance of folic acid supplementation.

“I was told to take iron and calcium. So, I took these medicines, but I don't know about the folic acid.” (woman in private hospital – P1, FGD1).

Interprofessional collaboration was suggested as a potential means of improving awareness, such as pharmacists providing information when women attend for pregnancy tests. Staff emphasized the heavy demand on their hospitals, suggesting the importance of maximizing the potential of other health sources.

“A pharmacist helps a woman with urine test for Rs. 50. He/she can advise women to have folic acid at that time also. They don't even say that. All they do is sell their pregnancy test kit and even when a case is positive say nothing… The main issue here is awareness.” (doctor in referral hospital – P2, FGD2).

## Discussion

### Principal findings

We found that although ANC has greatly improved in Nepal, additional focus is needed on care in the first trimester. Limited awareness of the importance of early ANC was shown to drive the trend of initiating ANC only after the first trimester. Women being unaware of their pregnancy and of the free ANC services available contributed to this. The influence of people surrounding the pregnant women guided their decision to only initiate ANC after the first trimester. Failures to achieve timely ultrasound scanning and effective supplementation, especially of folic acid, were described as key missed opportunities. Factors suggested to contribute to this were cost and lack of awareness of the importance of the timing of these actions in the early weeks of pregnancy.

### Results

This study adds to a growing body of literature on the timing of ANC initiation.^7^^,^^11^, ^12^, ^13^ The finding of the key role of limited awareness is consistent with recent studies conducted in Ethiopia and Tanzania^12^^,^^13^ that suggest level of knowledge as key to determining ANC timing. The significant influence of family is corroborated by another study conducted in Nepal,^14^ which suggested a key role of mothers-in-law in ANC decision-making and the role of limited knowledge in cases where mothers-in-law do not support ANC.

In recent years, great strides have been made in improving maternity care in Nepal through community awareness. Previous studies have reported women's groups^15^ and exposure to media^16^^,^^17^ as driving factors for increasing ANC uptake. There has been high-level political commitment by the Nepalese government, with investment in free maternal health services and incentives to encourage attendance at 4 ANC visits and skilled attendance at birth. However, the findings of this study highlight how there is still poor knowledge of the appropriate timing of ANC services, including ultrasound scanning. Timely ultrasound scanning enables accurate gestational dating,^7^ and detection of multiple pregnancies and major fetal abnormalities.^18^ This allows for early risk stratification and appropriate management. Ultrasound services were also suggested as a means of attracting women to ANC. This is corroborated by a scoping review of point-of-care ultrasound in underresourced countries.^19^

Folic acid is sometimes provided after the first trimester in Nepal in situations such as that of our previous study, in which 85% of women were offered supplements at their first visit, but only 22% attended during their first trimester.^8^ Supplementation should be started as soon as possible, preferably 4 weeks before conception.^20^ Nepal suffers from a high prevalence of neural tube defects, suggested by Bhandari's study^21^ to be 4.0 per 10,000 for selected neural tube defects. This can be improved by effective folic acid supplementation, which can improve birthweight and reduce the risk of neural tube defects and neonatal mortality.^21^^,^^22^

This study mentioned the issues of the cost of services and poor awareness of the free health services provided by the Nepalese government. Economic factors are key obstacles to ANC utilization in low-resource countries.^23^ Poor awareness of free services is compounded by inequalities, which further marginalize groups in rural areas.^24^^,^^25^ Although inequalities in ANC uptake have not been explicitly explored in this study, they have received considerable attention elsewhere.^17^^,^^26^ The literature presents various factors associated with late initiation of ANC, often after the first trimester. These include high travel time,^27^ low education, high parity, poverty,^28^ unintended pregnancy,^29^ and intimate partner violence.^30^ High travel time may be particularly relevant to Nepal, where there are logistical difficulties in reaching ANC services from remote areas. Living in more remote and rural areas is associated with late ANC, with only 58% of women in rural areas receiving ANC in the first trimester as opposed to 71% of women in urban areas.^1^

### Clinical implications

This study highlights the need for supporting policy change with education campaigns to increase awareness of the importance of ANC in the first trimester among women and their families and communities. This would address the key factors behind attendance after the first trimester and help improve the missed opportunities of effective ultrasound scanning and supplementation. This could have widespread benefits for health in Nepal. For example, in the context of a low-income country with limited health resources, infants with congenital defects place an additional burden on families and society. Shifting the focus to ensuring the appropriate timing of ANC would help minimize these missed opportunities.

### Research implications

This study has made apparent the need to raise awareness of the importance of early ANC among women and their communities. It demonstrates that advice from people around the pregnant woman resulted in attendance to ANC only after the first trimester, but further research is needed with these groups to understand their reasoning and determine the most effective way of achieving appropriate education to support the policy of early ANC. Education, reducing costs, and interprofessional collaboration were also identified as ways to minimize missed opportunities. Research into creating effective interventions to increase timely attendance, folic acid uptake, and targeted ultrasound scans should be conducted.

### Strengths and limitations

This study included a diverse range of staff members and stakeholders at all 3 levels of Nepal's health system, thus providing considerable insight into ANC in Nepal. However, time and financial constraints limited the study to Kathmandu and Makwanpur, omitting the most remote areas of Nepal where adequate care is challenging. This limitation was partially alleviated by the inclusion of staff who had previously worked in remote areas and stakeholders who have a broader understanding of ANC in Nepal. Furthermore, the topic guide used in focus groups asked about general delays in ANC, which often elicited comments about delays in early pregnancy, but could have been more focused on addressing the first trimester specifically. In examining missed opportunities, most of the information was provided by staff rather than the women utilizing ANC. This may be because the women are less accustomed to giving their opinions on such subjects, or because of greater disruption in these focus groups because of the presence of their infants, potentially contributing to less fruitful discussions. This resulted in insufficient data from women to substantiate some of the narratives of the health providers when referring to their own actions or those of women. In addition, through recruiting women from the postnatal wards, the perspectives of women who gave birth at home were missed. These unrepresented women may be most affected by the themes identified, such as family opposition to ANC, and may be particularly susceptible to missing opportunities of ANC in the first trimester.

## Conclusions

In this study, we found several missed opportunities associated with not attending ANC in the first trimester. The most notable were the benefits of early ultrasound scanning and folic acid supplementation. Many factors influenced the frequent attendance of ANC only after the first trimester. This study suggests that such timing is driven by limited awareness of the importance of early ANC among women and their families and communities. Therefore, although alleviating costs associated with ANC and enhancing interprofessional collaboration were identified as potential ways of improving ANC, education is crucial to targeting ANC in the first trimester specifically. Improving awareness of the importance of early ANC is needed among women, their families, and the community as a whole. Only then can the full potential of timely ANC be realized for enhancing maternal and neonatal outcomes.

**Table 4:** Stakeholder interviews

Departmental clinical leadership	(Former) presidents of national societies related to maternal health	Roles in the department of health	Health coordinator
6	3a	2	1

^a^Two of whom overlap with the head of department category.

**Table 5:** Illustrative quotes from the 4 themes identified in participant responses and the relevance of limited awareness to each of these

Theme	Quotes
Importance of awareness	• “They should inform us because we aren't aware of all these (pregnancy-related) things. We will come to the hospital because of some problems and they have to make us understand.” (Woman in private hospital – P3, FGD2) • “There are also cases where women only find out about their pregnancy when the baby starts moving.” (Nursing staff in secondary hospital – P4) • “…ANC package would be better if it was decided to be before 4 months to identify complicated cases and confirm pregnancy properly. If first ANC visit is made before 4 months, that is 16 weeks, the identification of disabled babies can be done and those cases can be managed easily.” (Nursing staff in secondary hospital – P4) • “So, I think we should improve awareness among people. There is the requirement of public awareness.” (Doctor in private hospital – P2, FGD2)
Influence of family and community	• “The neighbors shared about ANC checkups and that's when we went for the checkup.” (Woman in secondary hospital – P3, FGD3) • “Mothers-in-law are found saying, ‘We gave birth on our own. Everything is fine and once the baby starts moving, you can go to the health institute then.’” (Doctor in referral hospital – P4, FGD2) • “Some of them even visit the hospital after completion of 3 months when they come to feel the movement of the fetus. When we ask them the reason, they say that it was a matter of family.” (Doctor in private hospital – P1, FGD2) • “They listen to their friends. They say, ‘I was told to go only after 4 months.’” (Doctor in private hospital – P6, FGD2) • “First, the people did not know about the pregnancy checkup. Then, they go for the ANC checkup, everyone talks about the ANC checkup, and this is the change.” (Woman in secondary hospital – P7, FGD2) • Limited awareness: “Awareness is the key. Not only the patient but also her family and people around her should be aware. Patients only come after 3 months, and when we ask why they have only visited us now, they say that their near ones only came when they were 3 months pregnant. So, they advised them to do so as well. Women, instead of deciding themselves and using logic, follow others’ advice. And the ones who are giving advice are giving wrong advice. This is the reason why the society as a whole needs to be made aware.” (Doctor in referral hospital – P4, FGD2)
Importance of ultrasound	• “There should be good doctors, ultrasound machines and all the other services should be managed.” (Woman in secondary hospital – P3, FGD 3) • “The main reason behind the mortality of delivering women today is the delayed arrival of patients to health institutions. The ones who come to us in time are handled properly and they do not face more complications. But the ones who come during delivery only or visit us only once or have not had a single ultrasound scan are more likely to face complications.” (Doctor in referral hospital – P6, FGD2) • “In the 4 visit protocols, visits in the first trimester are not mentioned. The first visit should be made as soon as the mother knows she is pregnant, in the first trimester. This must be done to ensure that the implantation is at the correct place. In 1 in every 100 pregnancies, there is chance of ectopic pregnancy. This must also be addressed at first. This has not been covered in the current antenatal visit schedules.” (Stakeholder 10) • “If the government wants to invest, they have to make ultrasound free too. If 1 ultrasound at 20 weeks of gestation is provided free of cost, this can also attract women to do ANC checkups.” (Stakeholder 3) • **Limited awareness:** “They say that they will be diagnosed with ultrasound only after 3 months. So, they have to visit only after 3 months.” (Doctor in private hospital – P6, FGD2)
Concern about neural tube defects	• “We don't get iron tablets on time, so it should be provided and managed.” (Woman in secondary hospital – P2, FGD2) • “For those who plan their pregnancy, folic acid must be given before pregnancy because congenital abnormalities are increasing and must be prevented.” (Stakeholder 3) • “Next is that government must provide folic acid for free. Only iron is provided free. Although the iron and folic acid are mixed, it is only given from the second trimester. So, for the first trimester, folic acid is necessary.” (Stakeholder 3) • “If their insurance policy covers every expense, patients will come beforehand, maybe as early as 3 months before pregnancy. This way preconception counseling can be conducted as well.” (Doctor in referral hospital – P5, FGD1) • **Limited awareness:** “The next thing is that there is very minimal knowledge about preconception counseling… The preconception counseling is usually provided only to the women who have history of subfertility.” (Doctor in private hospital – P3, FGD2)

*ANC*, antenatal care.

**Table 6:** Data regarding the timing of first antenatal care contact and ultrasound from a recent service evaluation in the same 3 hospitals^8^

Contact	Private hospital	Secondary hospital	Referral hospital
% (n) who attended a visit in the first trimester	49% (34)	27% (26)	15% (57)
Wk of gestation at first contact Median (IQR)	13 (8−17)	16 (13−16)	20 (15−26)
Ultrasound scan before 24 wk (WHO only)	81% (56)	27% (26)	52% (191)

*IQR*, interquartile range; *WHO*, World Health Organization.

## Appendix Supplementary materials

Image, image 1

## Footnotes

Supplementary material associated with this article can be found in the online version at doi:10.1016/j.xagr.2022.100127.
