# Supplementary figures and images for: Antenatal care in Nepal: a qualitative study into missed opportunities in the first trimester
Source: AJOG Glob Rep. 2022 Nov 4;2(4):100127. doi: 10.1016/j.xagr.2022.100127 (PMC9703804; doi:10.1016/j.xagr.2022.100127)

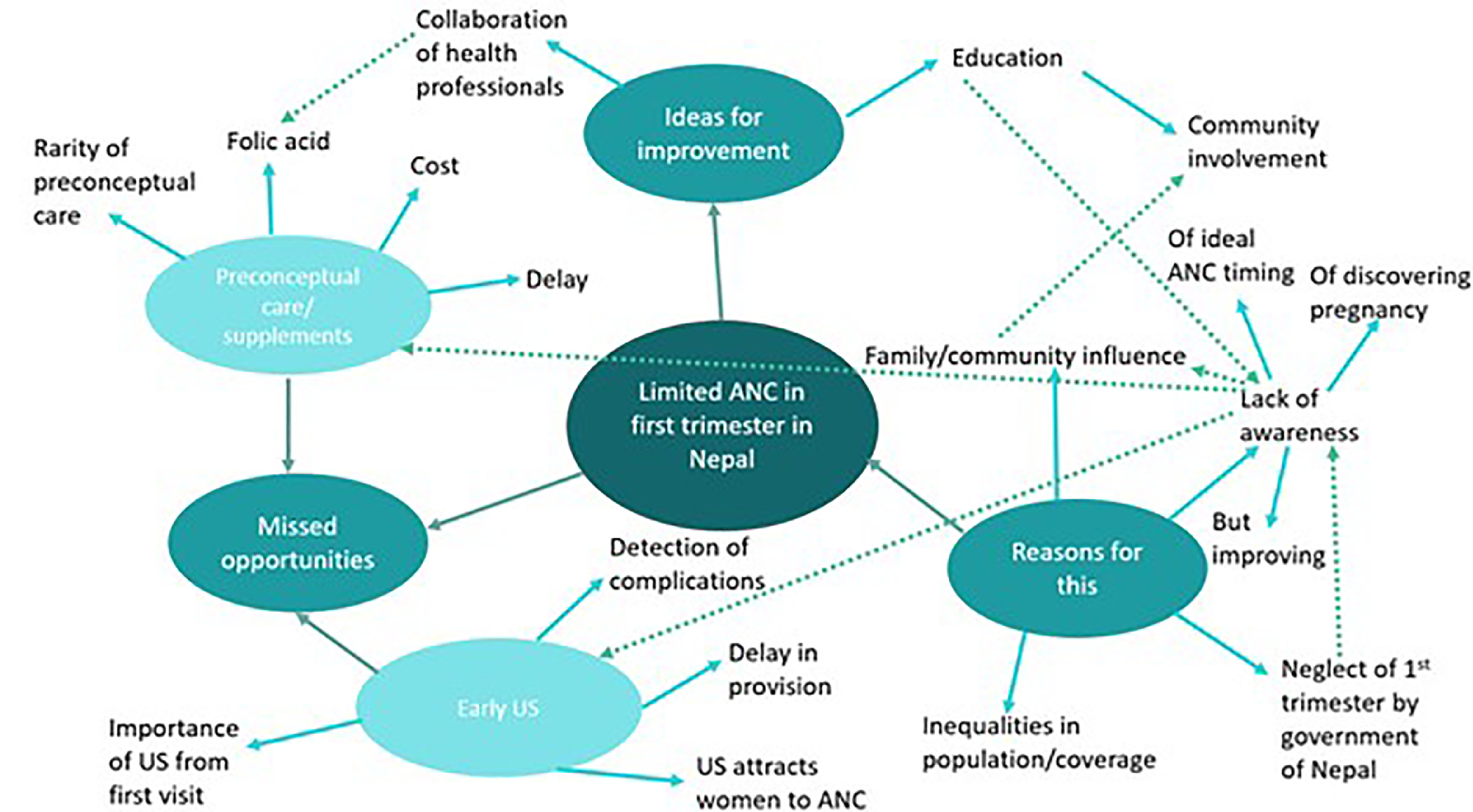

Supplement: Supplementary file 1 [file mmc1.jpg]
